# Efficient method for comprehensive computation of agent-level epidemic dissemination in networks

**DOI:** 10.1038/srep40885

**Published:** 2017-01-20

**Authors:** Gilberto M. Nakamura, Ana Carolina P. Monteiro, George C. Cardoso, Alexandre S. Martinez

**Affiliations:** 1 Universidade de São Paulo (USP), Faculdade de Filosofia, Ciências e Letras de Ribeirão Preto (FFCLRP), Av. Bandeirantes 3900, Ribeirão Preto 14040-901, Brazil; 2 Instituto Nacional de Ciência e Tecnologia - Sistemas Complexos (INCT-SC), Rio de Janeiro 22460-320, Brazil.

## Abstract

Susceptible-infected (SI) and susceptible-infected-susceptible (SIS) are simple agent-based models often employed in epidemic studies. Both models describe the time evolution of infectious diseases in networks whose vertices are either susceptible (S) or infected (I) agents. Precise estimation for disease spreading is one of the major goals in epidemic studies but often restricted to heavy numerical simulations. Analytic methods using operatorial content are subject to the asymmetric eigenvalue problem, limiting the use of perturbative methods. Numerical methods are limited to small populations, since the vector space increases exponentially with population size *N*. Here, we propose the use of the squared norm of the probability vector to obtain an algebraic equation, which permits the evaluation of stationary states in Markov processes. The equation requires the eigenvalues of symmetrized time generators and takes full advantage of symmetries, reducing the time evolution to an *O(N*) sparse problem. The calculation of eigenvalues employs quantum many-body techniques, while the standard perturbation theory accounts for small modifications to the network topology.

One of the main goals in epidemics studies is to correctly predict the time evolution of a given disease within a population^1^. The forecasting procedure, which may take numerical or analytical formulations, often encounters obstacles due to heterogeneous populations and the limited knowledge of the disease spreading dynamics. For instance, ambiguous symptoms among distinct diseases may under or overestimate total reported infections, leading to incorrect estimates of transmission rates. Several epidemic models have been tailored to better grasp general behaviours in disease spreading^2^,^3^. Among them, the simplest one is the susceptible-infected-susceptible (SIS) model. The SIS model is a Markov process and describes the time evolution of a single infectious disease in a population formed by susceptible (S) and infected agents (I). The infected agents carry the disease pathogens and may transmit them to susceptible agents with constant transmission rate *β*. The model also contemplates cure events for infected agents with constant cure rate *γ*, introducing competition between cure and infection events.

There are two approaches often employed to mimic the disease spreading dynamic in populations with fixed size *N*: compartmental and stochastic ones^4^. In the compartmental approach, relevant properties derived from either infected or susceptible agents are well-described by averages, a direct result from the random-mixing hypothesis^5^,^6^. This enables one to derive non-linear differential equations to match the evolution of disease throughout the population. For instance, the number of infected agents in the compartmental SIS model, *n(t*), satisfies the following differential equation:


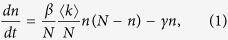


with 〈*k*〉 = *N* − 1 and *R*_0_ = *β*/*γ* is the basic reproduction number^7^. For homogeneous populations this is the expected behaviour. However, real agents differ from each other, leading to a heterogeneous population, in disagreement with the random-mixing hypothesis^8^. Stochastic approaches may also be further classified according to their descriptive variable. Similar to the compartmental model, the mesoscopic interpretation usually describes the time evolution of global variables^9^,^10^; in addition, it allows for time fluctuations. Adding another layer of detail, the microscopic approach describes disease evolution of individual agents depending on their connections, introducing time fluctuations at the agent level.

Central to the microscopic stochastic approach is the underlying network used to reproduce the heterogeneity typically found within populations^5^. In the network scheme, agents are represented by vertices and their connections are distributed according to the adjacency matrix *A* for the assigned network configuration^11^. In this case, it is well-accepted that the mean degree 〈*k*〉 in Eq. (1) describes the averaged process^3^. Different from the random-mixing hypothesis, non-trivial topological aspects of *A* may be incorporated in the effective transmission and cure rates, producing complex patterns in epidemics^3^. The time evolution is dictated by a transition matrix 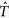, whose matrix elements *T*_*μν*_ are transition probabilities from network configuration *ν* to *μ*^12^. Typically, one assumes Markovian behaviour to describe disease transmission and cure events, in accordance with the Poissonian assumption^3^. The difference between the compartmental and stochastic schemes leads to distinct evolution patterns for the statistics as well. For instance, Eq. (1) displays stable infected population for *γ* < *β*, power-law behaviour for *γ* = *β* and exponential decay of the infected population otherwise. While all three behaviours are also observed in stochastic approaches, fluctuations become much more relevant when the number of infected agents, 〈*n(t*)〉, is small compared to total population, *N*. Incidentally, this is the relevant regime to sanitary measures and health policies to contain real epidemics in early stages.

The Markovian approach produces accurate results if the infection transmission mechanism is known ref. 3. Its usability has been restricted insofar to numerical simulations with small *N* since the number of available states is 2^*N*^ and 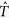 is generally asymmetric^12^. Therefore, left- and right-eigenvectors are not related by transpositions, limiting the exact diagonalization to small values of *N* or special transition matrices. However, recent advances in the area by Van Mieghem *et al*.^13^,^14^ have shown that, in addition to spectral results, adequate local mean-field approximations (*N*-intertwined mean-field approximation) still carry over the heterogeneity of the contact network of agents. In ref. 13, the authors demonstrate the stationary state of SIS model is disease free if *R*_0_ is greater than the largest eigenvalue of *A*. Subsequent studies addressed the effects of network perturbations to the epidemic threshold^15^,^16^,^17^,^18^. One of the goals in epidemic studies is the ability to correctly predict how small parameter or topological changes in the network affect disease spreading. If such predictions are robust, preemptive actions to lessen the epidemic are expected to achieve better results^19^. Small changes are exactly the subject of perturbation techniques, which make extensive use of scalar product between left- and right-eigenvectors. In epidemic models, however, one must deal with asymmetric transitions, prohibiting perturbative schemes based on normed scalar products.

Here, we devise a method to avoid difficulties related to the asymmetry of 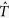 using the squared norm of the probability vector, |*P(t*)|^2^, that can be physically interpreted as the exponential decay of the quadratic Rényi entropy. We discuss the mathematical aspects concerning the agent-based SIS model, with emphasis on the operatorial content and symmetries. The time evolution equation for |*P(t*)|^2^ is obtained, and it is shown to depend only on eigenvalues and eigenvectors of the symmetric time generator 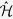. As occurs in Quantum Mechanics, symmetries bring the matrix representation of 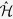 to block diagonal form, with each block labeled by equivalents of quantum numbers, thus reducing the complexity of the spectral analysis. Furthermore, the differential equation for |*P(t*)|^2^ simplifies to an algebraic equation for stationary states or transient states with maximum Rényi entropy. The solutions obtained in this way are valid for general Markov processes and preserve agent-agent correlations, usually neglected under mean-field approximations. In addition, the proposed method allows for seamless reproduction of traditional perturbative results, shedding light on the role played by perturbations in epidemics. Results obtained using perturbative methods for epidemics in agent-based networks for SIS model are shown and the main conclusions are stated.

## Transition matrix

**Figure 1:**
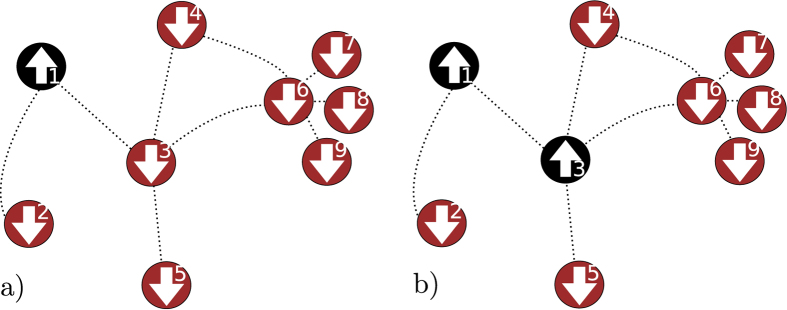
Notation for agent configurations in epidemic process. In (**a**) agent at vertex *k* = 1 is infected. The graph configuration is 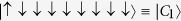. In (**b**) a second agent is infected at *k* = 3, leading to the configuration 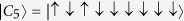.

The transition matrix 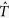 evolves the health states of *N* agents along time. Since the time evolution deals with the transmission of pathogens among agents, the construction of 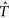 must carry information about the network of contacts between them. This is done by employing graphs, which are mathematical realizations of networks^11^,^20^. They are formed by a set of interconnected vertices *V*_*k*_ (*k* = 1, …, *N*). The connections are described by the adjacency matrix *A (N* × *N*), whose matrix elements are either 0 or 1. Vertex *k* is connected to vertex *k*′ if *A*_*kk*′_ = 1 and 0 otherwise. Within our framework, each vertex *V*_*k*_ holds a single agent, whose current status is identified by *σ*_*k*_. The variable *σ*_*k*_ may acquire two values, namely, *σ*_*k*_ = ↓ (susceptible) or *σ*_*k*_ = ↑ (infected), fulfilling the two-state requirement. The configuration 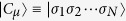, with *μ* = 0, 1, …, 2^*N*^ − 1, describes any agent state in the graph. As illustrated in Fig. 1, we follow^16^ and enumerate the configurations using binary arithmetic: 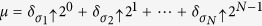, where the Kronecker delta 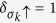 if *σ*_*k*_ = ↑ and null otherwise. The set {|*C*_*μ*_〉} spans a discrete Hilbert space 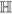. For clarity’s sake, we use the following notation: Latin indices run over vertices 1, …, *N*, while Greek indices enumerate 2^*N*^ configurations in 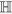.

The next step required to build the transition matrix is the definition of operators and their actions on vectors in 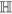. For that purpose, the transitions described in epidemic models must first be broken down into simpler operators, whose actions over the configurations |*C*_*μ*_〉 possess equally simple interpretation. These operators express the most basic transitions among different health states for each agent as well as measure their current health state. For this task the 1/2-spin lowering and raising operators serve as inspiration since they also describe transitions among different quantum numbers of quantized angular momentum^21^. For instance, the operator 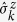 is set to probe whether the agent located at vertex *k* is infected (↑) or not (↓):


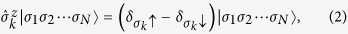


while the operator 
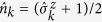
 extracts the number of infected at vertex *k*. Accordingly, the operator 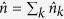 extracts the total number of infected agents in the population. The *k*-th agent health status is reversed by action of operators 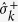 and 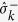:


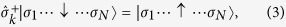



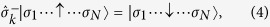


null otherwise. The localized operators 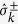 and 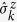 satisfy additional algebraic properties. For each *k*, the set 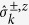 forms a local su(2) algebra, 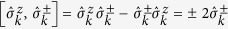, 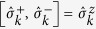. Note that according to this definition, the operators 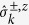 produce *local* fermionic anticommutation relations 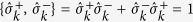, 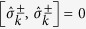, *i.e*., they behave like fermions at same vertex. However, their non-local algebraic commutation relations are bosonic: 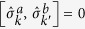, for *k* ≠ *k*′ and *a, b* = ±, *z*. This means the action of two localized operators, from different vertices, independs on the order of operators themselves, 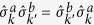. Although outside the scope of this paper, for completeness’ sake, we add that the mixed operatorial nature of 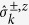 must be taken into account if solutions by Fourier transforms are pursued, as learned from condensed matter problems^22^,^23^,^24^.

For any Markov process, the transition matrix 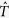 holds the probabilities of allowed transitions among configurations |*C*_*μ*_〉, for a fixed time interval *δt*. Let *P*_*μ*_(*t*) be the probability to find the system in the configuration |*C*_*μ*_〉 at time *t*. The collection of all *P*_*μ*_(*t*) forms the probability vector,


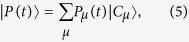


with 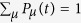. In short, |*P(t*)〉 is the description of the system with *N* agents and the time evolution is simply


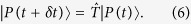


Notice, however, that 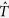 depends intrinsically on the value of *δt*: for a very large *δt*, the transition matrix must contemplate both simple and complex transitions. By simple transition we mean a transition that details a single change in the health state of a single agent, whereas complex transitions may express several changes in health states for one or more agents. As a result, simple transitions are modelled after one- or two-agent operators whereas complex transitions require multiplicative composition of one- and two-agent operators. For instance, 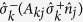 outlines the disease spreading from the *j*-th agent to the *k*-th agent, followed by its recovery. As *δt* increases, the number of distinct transitions also increases and, hence, one would have to evaluate the likelihood corresponding to each transition.

A much easier approach takes small 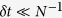, ensuring that only one- and two-agent transitions are relevant, with transition rates *γ* and *β*/*N*, respectively. The reason behind this statement lies in the way the transition probabilities are evaluated in general: by hypothesis, the transitions between *individual* states are assumed to be well-described by independent Poisson processes with constant rates^3^. One transition is observed in a time interval *δt* for a system with *N* agents, on average, if *δt*[*N(β* + *γ*)] = 1. Hence, if 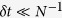, two or more transition events become less likely, and one- and two-agent operators are the only relevant ones. It is important to remark the discussion above concerns the time evolution expressed by the Markov process with real time evolution. In numerical simulations of epidemic spreading, time steps that (not) scale as *O(N*^−1^) lead to contact-processes (reaction-processes)^25^. If 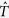 as a contact-process is known, then 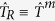 is the transition matrix in the reaction-process with time interval 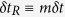. Thus, the reaction-process evolves faster over time than the contact-process. Here, we explicitly assume the Poisson hypothesis to estimate the transition probabilities *β* and *γ*, considering either a single cure or single infection event during a time interval *δt*.

**Figure 2:**
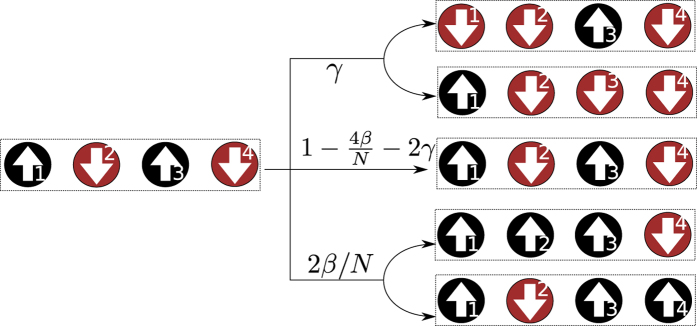
Markov process for *N* = 4 in a complete graph. At time *t*, the probability to find the system in the configuration 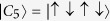 is *P*_5_(*t*). Configuration |*C*_5_〉 may evolve into five distinct configurations, after the time interval *δt*, due to disease transmission and recovery processes in the SIS model. If the infected agent at *k* = 1 recovers, then the resulting configuration is |*C*_4_〉. Similarly, if the infected agent at *k* = 3 recovers, then the resulting configuration is |*C*_1_〉. Both events occur with transition rate *γ*. Concerning disease transmission events, the infected agent at *k* = 1 (*k* = 3) may either transmit the pathogen to the susceptible agent at *k* = 2 or *k* = 4, resulting in the configuration |*C*_7_〉 or |*C*_13_〉. Since both infected agents produce the same resulting configurations, in the complete graph, the transition rate is 2*β*/*N*. The remaining transition contemplates the event in which agents neither recover or transmission the pathogen. In this case, the resulting configuration is |*C*_5_〉, with transition rate 1 − 2*γ* − 4*β*/*N*.

In the SIS model, any previously infected agent at vertex *k* evolves to one of three distinct outcomes during the time interval *δt*: infection of one connected susceptible agent; cure of agent at vertex *k*; or nothing happens and the system remains unchanged. The operator 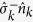 produces the desired cure action, while 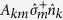 transmits the disease from the *k*-th agent to *m*-th agent, given the *k*-th agent is currently infected and the other is susceptible, as exemplified for the complete graph depicted in Fig. 2 (top and bottom processes); otherwise the states of the agents remain unchanged. Setting 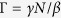, the corresponding transition matrix reads


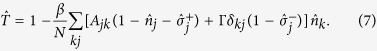


**Figure 3:**
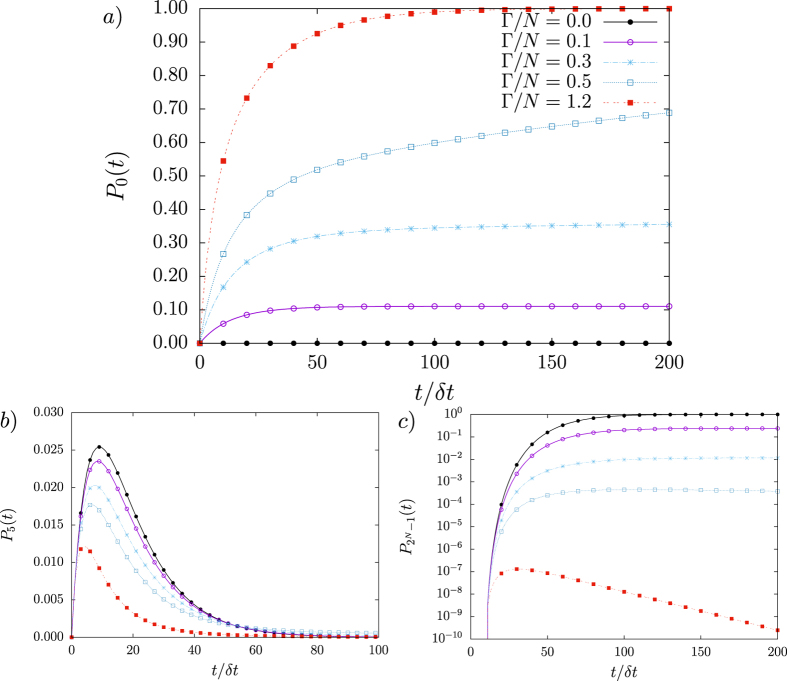
Configuration probabilities *P*_*μ*_(*t*) for *μ* = 0, 5 and 2^*N*^ − 1 with *N* = 12 in a complete network with initial condition *P*_1_(0) = 1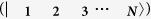. In (**a**) probability *P*_0_(*t*) to observe all-cured configuration at time *t* for various couplings 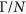. In (**b**) *P*_5_(*t*) refers to the probability of transient configuration 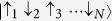, while (**c**) exhibits the probability with all-infected agents, 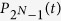, in log scale. The coupling 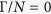 represents the SI model, whose stationary state is described by all-infected configuration. For 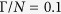, the stationary state is a linear combination of distinct |*C*_*μ*_〉, including all-cured |*C*_0_〉 and all-infected 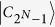 configurations. For intermediate couplings 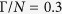 and 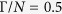, the stationary state is |*C*_0_〉 with large transient Δ*τ* ~ *o(N*^4^).

Brief inspection reveals 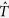 is asymmetric, implying the existence of distinct left and right eigenvectors. An example of the matrix representation of 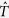 is shown in Supplementary Information: Appendix A. Even though 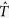 in Eq. (7) is valid only for SIS model, generalizations for more realistic epidemic models such as SIRS and SEIRS are available using the Weyl matrices^26^ (see Supplementary Information: Appendix B). Once the explicit action of 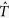 is known, *P*_*μ*_(*t*) are readily evaluated. Figure 3 exhibits numerical results for *P*_*μ*_(*t*) for *μ* = 0, 5, 2^*N*^ − 1, parameter 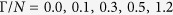, *P*_1_(0) = 1 as initial condition and *N* = 12, in a complete network. For increasing 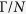, the probability *P*_0_(*t*) to find the system without infected agents also increases, while the opposite holds true for 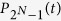, where all agents are infected. The probability for the intermediate configuration |*C*_5_〉 is also displayed to illustrate transient effects. Despite its simplicity, Eq. (7) produces a power-law behaviour, exemplified in Fig. 3 with 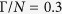, 0.5, in which the time interval to reach the stationary state |*C*_0_〉 is much larger than the total number of agents, 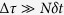.

Derivation of Eq. (7) considers a single network realization. If an ensemble containing *M* graphs is considered, properly sampling the network, the only modification required is the following: 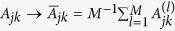. The reason is the following: networks only assign distribution rules for connections, leaving the vertex distribution and, therefore, the Hilbert space unchanged. For each graph *l* = 1, …, *M* in the ensemble, one applies the associated transition matrix 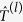 on the initial configuration |*P*^(*l*)^(0)〉 producing the probability vector |*P*^(*l*)^(*δt*)〉. In this way, one must also consider the ensemble averages. In particular, the average probability to find the system in configuration |*C*_*μ*_〉 is 
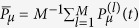
. Since the procedure is equivalent to the average of 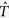 over the graph ensemble – the network sample – one needs only to consider the network distribution of *A*. For clarity, we drop the bar symbol and always assume the average over graph ensemble.

## Squared Norm

Any transition matrix 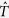 carries two operatorial contributions: the diagonal part tell us the likelihood the system remains unchanged after a time interval *δt*, whereas the off-diagonal part reveals the available outcomes and their corresponding probabilities. In our assumptions, *δt* is small enough to enforce that at most one local change may occur during the time interval *δt*. This assumption allows us to rewrite the transition matrix in a more convenient way: 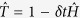. By substituting this expression for 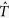 in Eq. (6), one arrives at 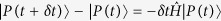. Now, if all *P*_*μ*_(*t*) are continuous functions in time, then the following power series converges: 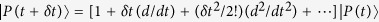, in the continuous-time Markov process. Therefore, up to *O(δt*^2^), the time evolution is given by 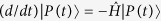. In general, the probabilities *P*_*μ*_(*t*) obey the following system of differential equations,


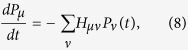


where the matrix elements 
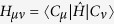
 are either negative for allowed off-diagonal transition rates or negative for diagonal positive rates. The compartmental equation^3^,^5^,^27^ is derived from Eq. (8) by neglecting dispersions among agents, whereas the *N*–intertwined mean-field approximation^14^,^18^ for SIS model is also a result from Eq. (8) with the assumption the local averages 〈*n*_*k*_(*t*)〉 are independent variables (see Supplementary Information: Appendix C).

Due to the striking similarity with Schrödinger equation^21^, 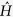 is often interpreted as the Hamiltonian – the time generator – of the Markov process. However, unlike the Hamiltonian of quantum theories, 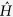 is real and, usually, an asymmetric operator. As a result, there exists one right-eigenvector |*ϕ*_*μ*_〉 and one left-eigenvector 〈*χ*_*μ*_| for each eigenvalue *λ*_*μ*_ of 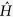, with 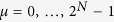. Although |*ϕ*_*μ*_〉 and 〈*χ*_*μ*_| are not related by Hermitian conjugation, they decompose the identity 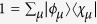 (completeness) and are orthogonal to each other 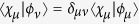. Furthermore, *λ*_*μ*_ ≥ 0 for any *μ* since 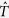 is positive definite.

**Figure 4:**
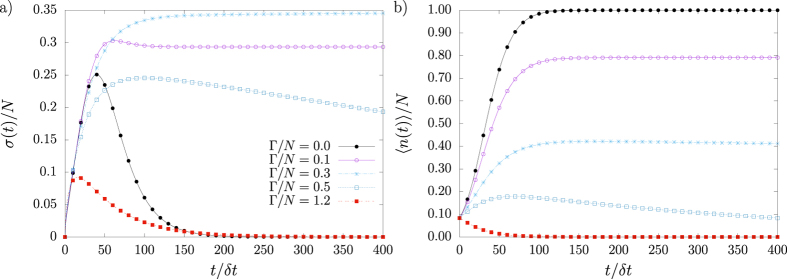
Standard deviation *σ(t*) and mean number of infected agents 〈*n(t*)〉 for SIS model with *N* = 12 in the complete network. The statistics *σ(t*) and 〈*n(t*)〉 are shown in (**a**,**b**), respectively. Intermediate cure/infection rates 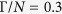 and 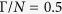 eradicate the disease after very large time intervals: *σ(t*) exhibits initial rapid growth, develops a maximum at 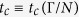 and then decays as power-law.

The formal solution for Eq. (8) reads 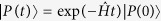, as long as 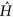 is time independent, *i.e*., the rates *β* and *γ* remains unchanged along time. In terms of left- and right-eigenvectors, 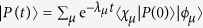. As a result, only the modes with vanishing eigenvalues *λ*_*μ*_ = 0 survive for large *t*. These modes are the stationary states^12^. Statistics for a given observable 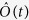 are calculated according to 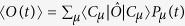. Among the relevant observables in disease spreading models, the mean number of infected agents, 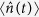, and variance, *σ*^2^(*t*), both exemplified in Fig. 4, are often relevant variables. Formally, they admit eigendecomposition: 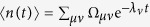 and 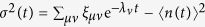, with 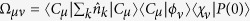 and 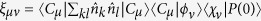.

While left- and right-eigenvectors are expected to decompose the identity, their actual computation is rather cumbersome, doubling the computational effort and being prone to convergence errors. They also lack a clear physical interpretation. Ultimately, quantitative understanding of fluctuations and heterogeneity in epidemics is severely affected by the way left- and right-eigenvectors are currently handled. For instance, investigations using group theoretical methods usually relies on a single set of eigenvectors, for a given representation. This means symmetries are often overlooked. As a result, agent-based models have provided limited insight about epidemics in general^3^, despite their relevance to the study of emerging infectious diseases^4^.

**Figure 5:**
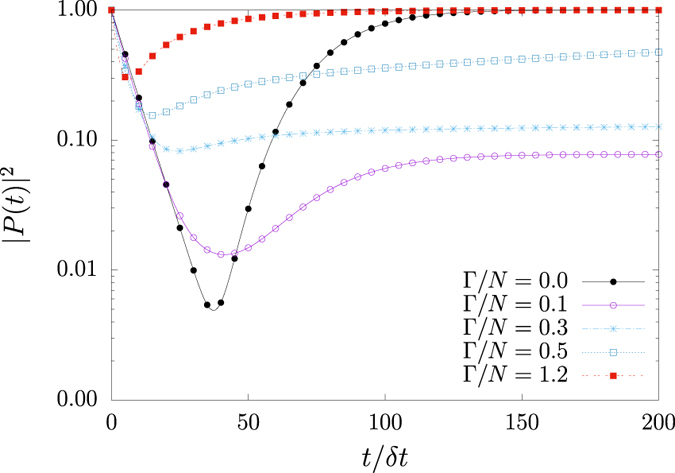
|*P(t*)|^2^: Sum of squared probabilities of infected agents in all possible configurations *μ*. SIS model with *N* = 12 and *P*_1_(0) = 1. For each coupling parameter 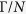, |*P(t*)|^2^ always develops a global minimum followed by constant value at stationary state, being unity only for single state configurations. Logarithmic scale is employed to emphasize extremal points at 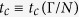.

A sensible way to overcome the problematic related to left- and right-eigenvectors is simply to avoid them altogether. One possibility is to consider quantities that preserves the scalar product. Here, we consider the squared norm, 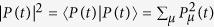, which remains invariant under unitary transformations. Notice the total probability conservation 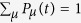 does not warrant |*P(t*)|^2^ conservation over time. For instance, examples are found in any Markov process that starts from an arbitrary, but otherwise unique, initial state and arrives to a single stationary state. In these particular cases, although |*P*(0)|^2^ = 1 and 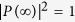, the squared norm of the probability vector during the transient period is |*P(t*)|^2^ < 1 if two or more states are available. This happens because the probability to observe the system in any transient state is now lower than unity. Therefore, |*P(t*)|^2^ changes over time, being a suitable candidate to represent the dynamics of the system. The epidemic models considered in this study, SIS included, fall into this category, as shown in Fig. 5.

The time derivative of |*P(t*)|^2^ is obtained by substituting Eq. (8) in 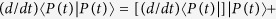

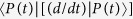
. Defining the Hermitian operator 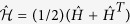, produces the following equation:


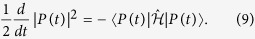


**Figure 6:**
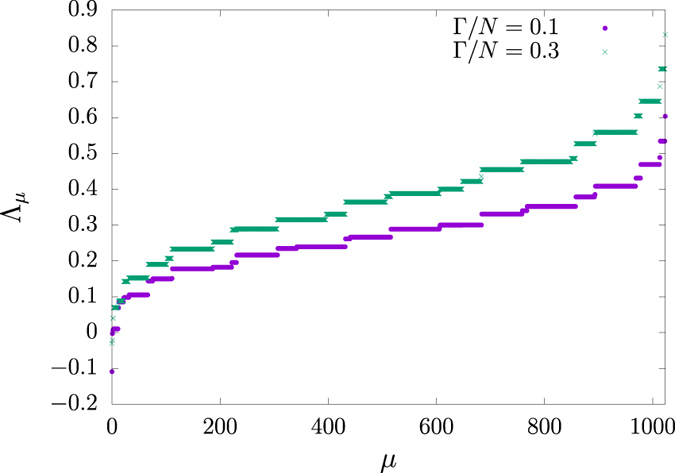
Sorted eigenspectra with *N* = 10 for the case of a complete network. Each filled circle (cross) represents one eigenvalue 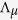 for coupling parameter 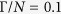 (0.3).

Unlike 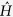, the operator 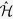 has eigenvalues 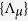 with corresponding left- and right-eigenvectors {*ψ*_*μ*_} related by Hermitian conjugation. The trade-off is that the eigenvalues 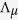 may assume negative values, as shown in Fig. 6, and the coefficients 
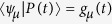
 are, in general, complex numbers. As a result, the coefficients *g*_*μ*_ are not probabilities. Despite this shortcoming, the coefficients *g*_*μ*_ are used to evaluate the probabilities *P*_*μ*_(*t*) for each configuration |*C*_*μ*_〉:


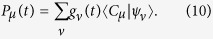


It is noteworthy that analytical and numerical diagonalization procedures are far more abundant for Hermitian operators than for asymmetric counterparts. However, the evaluation of *g*_*μ*_(*t*) as they are still poses as a hard problem. In what follows, we devise an alternative way to compute them for relevant time instants in the disease spreading process.

Using the completeness 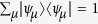 in the right-hand side of Eq. (9), one arrives at 
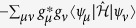
. When the same procedure is performed on the left-hand side of Eq. (9), the resulting expression reads


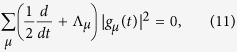


subjected to the constraint 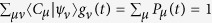. Now, Eq. (11) takes a simpler form if |*P(t*)|^2^ is constant, which is the expected outcome if the system reaches at least one stationary state. Under this assumption, Eq. (11) reads


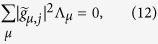


subjected to the constraint described above, and where the collection of coefficients 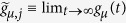 describes the *j*-th stationary state. Table 1 displays non-trivial solutions of Eq. (12) for 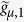 and 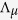 using the SI model with *N* = 3 and *β*/*N* = 0.1. *g*_*μ*_ are real numbers in this example chosen because it can be evaluated by brute force and tested against the correct answer. Of course, the trivial solution 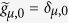 and 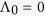 also satisfy Eq. (12).

**Figure 7:**
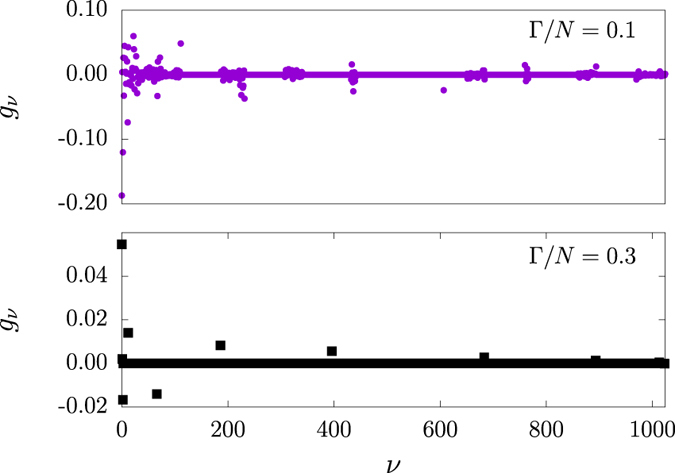
Solutions *g*_*ν*_(*t*_*c*_) in a SIS model with *N* = 10. The coefficients *g*_*ν*_ are the weighs used to calculate *P*_*μ*_ using Eq. (10). Non-trivial solutions to Eq. (12) are shown for 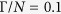 (top), 0.3 (Bottom). The coefficient distribution greatly differs depending on coupling parameter 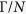.

In addition to stationarity, |*P(t*)|^2^ may also assume extrema values at time instants *t*_*c*_, leading again to Eq. (12), the difference being only the evaluation of coefficients *g*_*μ*_(*t*) at *t* = *t*_*c*_. Numerical examples are shown in Fig. 7. The times *t*_*c*_ are important for dynamics as the extrema of |*P(t*_*c*_)|^2^ inform us when disease spreading reaches critical moments. Accordingly, *t*_*c*_ may also be used to estimate the maxima for narrow peaked statistics. During the transient, the variance 〈*n*^2^(*t*)〉 − 〈*n(t*)〉^2^ is well described by a narrow function with peak near *t*_*c*_. The estimation improves as *N* increases. Therefore, by solving the constrained algebraic Eq. (12), either directly or via functional minimization, one evaluates the coefficients *g*_*μ*_, which in turn are used to evaluate the probabilities *P*_*μ*_ in Eq. (10). Because solving algebraic equations are far less demanding then solving sets of coupled differential equations, the behavior of |*P(t*)|^2^ dramatically improves the usability of agent-based models in the study of disease spreading.

Equations (11) and (12) introduce a novel way to tackle stochastic problems: asymmetric time generators 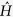 are replaced by symmetric operators 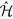, whose eigenspectra are used to assemble Eq. (12). The probabilities *P*_*μ*_ for each configuration of *N* agents are evaluated according to Eq. (10), provided the coefficients *g*_*μ*_ solve Eq. (12). Once the eigenvalues and eigenvectors of 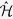 are known, the above procedure is simply the resolution of a single algebraic equation. However, the eigenvalue problem of *N* interacting agents is far from trivial. Since 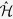 is a Hermitian operator, one may take advantage of well-known methods to solve the corresponding eigenvalues and eigenvectors. Among the now available methods, we point out the techniques from Hermitian many-body theories, largely employed in research fields such as quantum optics and condensed matter. These areas make heavy use of symmetries, reducing the computational efforts and also providing new insights about the fundamental properties and patterns in the system. In passing, we also remark that the number of symmetries of 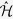 may differ from those found in the operator 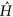. Furthermore, the stationary states or extremal solutions in the transient period carry the network topological information, as the adjacency matrix determines the eigenvalue distribution of 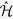. In the large 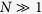 regime, the eigenspectrum 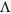 becomes dense o closely spaced. For completeness sake, we briefly discuss this regime in Supplementary Information: Appendix D.

Before moving on to applications, we address the physical interpretation of |*P(t*)|^2^. One important aspect from Eq. (9) concerns the fact that the global quantity |*P(t*)|^2^ acquires a dynamical meaning. As a result, any interpretation of |*P(t*)|^2^ at the time *t* should also be valid for other time *t*′ > *t*. Therefore, we restrict our analysis to a single time instant *t*. Under these circumstances, the squared norm of the probability vector may be also written as 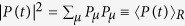, where 〈*P(t*)〉_*R*_ is an estimator for the average probability for each configuration at time *t*. For comparison purposes, we also define the standard estimator 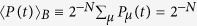, which is always constant. If all configurations are equiprobable at time *t*, then 〈*P(t*)〉_*B*_ is compatible with the Boltzmann hypothesis, from which one derives the entropy *S*_*B*_ = *N* ln 2. However, while 〈*P(t*)〉_*B*_ treats equally any configuration state |*C*_*μ*_〉, the estimator 〈*P(t*)〉_*R*_ distinguishes and weights them differently from each other. More importantly, the difference 〈*P(t*)〉_*R*_ − 〈*P(t*)〉_*B*_ is minimal when |*P(t*)|^2^ develops a minimum. As Figs 4(a) and 5 depict, the region near the minimum of |*P(t*)|^2^ is also the region where *σ(t*) develops a maximum. This evidence suggests |*P(t*)|^2^ plays a role similar to the entropy for the disease spreading stochastic process. In fact, several generalized entropies use |*P(t*)|^2^ in their definitions. For instance, Tsallis’ generalized entropy^28^ reads (non-extensive parameter *q* = 2): *S*_*T*_(*t*) = 1 − |*P(t*)|^2^. However, Eq. (9) is also valid for general extensive systems, hence, we discard *S*_*T*_(*t*). A more suitable choice for disease spreading processes is the quadratic Rényi entropy^29^,^30^:


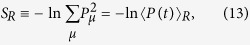


which is always extensive^31^. In what follows, we adopt the Rényi entropy *S*_*R*_ as the global quantity for epidemic models.

## Perturbation Theory

Perturbative methods are usually pursued because they provide a way to evaluate the effects of small modifications in the underlying model to measurable quantities, such as 〈*n(t*)〉. In the case of epidemics, there are two kinds of relevant quantities that affect the disease spreading dynamics, namely, the coupling parameters (transmission and recovery rates) and the adjacency matrix *A*. More precisely, perturbations in *A* means the statistics of *A* are changed by small but otherwise known amounts. Examples include the removal of 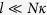 connections in a graph, where *κ* is the average degree for each vertex^11^, and reordering of a few connections among agents to produce a change in their clustering coefficients, 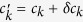, with 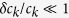. These perturbative schemes mimic sanitary and health measures often used to contain or control disease spreading.

Despite the relevance of perturbative theories, historically they have never enjoyed large endorsement in the study of epidemics. Common approaches include brute force computational power, but also perturbative theories for stochastic processes often relying on complex analyses, recurring to the formalism of Feynmann path integrals^32^. More recently, Wang *et al*.^18^ have used the Rayleigh-Schrödinger perturbation theory^33^ in the mean-field approximation, for the SIS model with coupled networks. The main advantage of their approach is that one can easily understand and evaluate the effects of topological perturbations. In this section, we use Eq. (12) to reintroduce the standard Rayleigh-Schrödinger perturbation theory for stationary states and states with maximum Rényi entropy during transient, without any additional approximation. In a sense, our method expands the ideas of Wang *et al*.^18^ while still accounting for correlations, usually neglected in mean-field approximations. Thus, corrections to measurable quantities are evaluated using standard algebraic methods and include any effect derived from fluctuations. As always, we focus on the SIS model to demonstrate the perturbative corrections, although we stress the procedure is valid for general Markovian epidemic models.

For the SIS model, the Hermitian time generator is 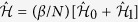, with


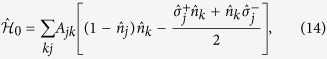



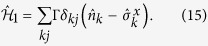


Here we will assume that the adjacency matrix elements *A*_*jk*_ are already averaged over an ensemble. As a practical application, we discuss the random removal of links from a complete graph as the network perturbation. This case is also convenient since the dynamical equations for 〈*n(t*)〉 are readily available from Eq. (8), whose solutions are known since all vertices are equivalent. However, the primary purpose of this application is to show the role of symmetries, which break down the initial problem into disjoint and smaller problems.

The complete network is obtained taking *A*_*jk*_ = (1 − *δ*_*jk*_). Despite its simplicity, this network provides relevant operatorial content. Defining the many-body spin operators as 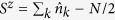, 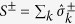, and 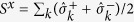, the resulting symmetrized time generator is


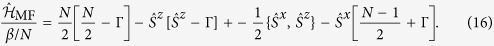


The operator 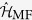 satisfies 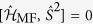, where 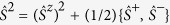 is the quadratic Casimir operator. Thus, the eigenvalues *s(s* + 1) are suitable labels, with *s* = *N*/2, *N*/2 − 1, … and *s* ≥ 0. This means, the operator 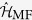 in Eq. (16) prohibits transitions among different *s*-sectors, *i.e*. 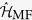 is block diagonal, each block with dimension *d* = 2*s* + 1. In addition, each block is also tridiagonal in the basis |*s, m*〉 (*m* = −*s*, −*s* + 1, …, *s*) as the 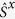 operator may only increase or decrease *m* by unity. Therefore, the largest *s*-sector block has, at most, dimension *d*_max_ = *N* + 1 and 3*N* − 2 non-null matrix elements, thus sparsity *O*(3/*N*). When both properties are considered, one realizes the *O*(2^2*N*^) computational problem has been reduced to *O(N*). Thus, symmetries considerations alone eliminate one of the main obstacles faced by agent-based epidemic models.

**Figure 8:**
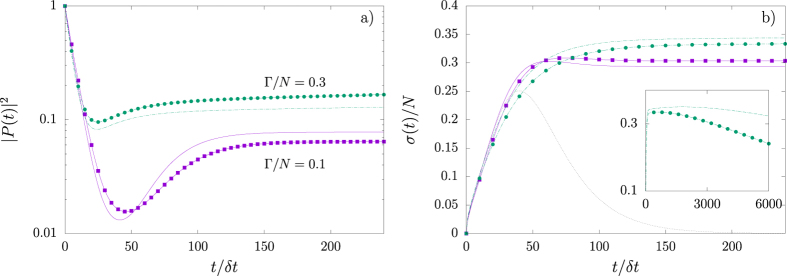
Perturbation in mean-field SIS model and *N* = 12. In (**a**) |*P(t*)|^2^ is plotted against time steps for SIS coupling parameter 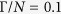 in the mean-field network *δp* = 0 (full magenta line) and in the perturbed network *δp* = 0.1 (magenta squares); the corresponding quantities are also shown for 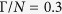 with *δp* = 0 (dashed green line) and *δp* = 0.1 (green circles). Topological perturbations decrease (increase) |*P(t*)|^2^ for 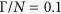 (0.3). In (**b**) *σ(t*) is plotted against time steps. The dotted line displays the expected SI behaviour for comparison. The inset shows *σ(t*) with 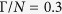 and *δp* = 0 (dashed green line) and *δp* = 0.1 (green circles) using increased time range. During transients, small perturbations *δp* may produce large modification to the statistics.

For general networks, the main strategy is to use the eigenvectors of 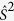 and treat any absent link among agents as perturbations. A simple perturbation to the complete network is obtained by considering a small probability 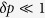 to independently remove links between vertices, *A*_*jk*_ = (1 − *δ*_*jk*_) (1 − *δp*). This procedure is equivalent to transforming the underlying network into a random network^20^,^34^, with connection probability 1 − *δp*. Perturbative effects to |*P(t*)|^2^ and *σ(t*) are shown in Fig. 8. Although both network and topological perturbations are simple, distinct perturbative effects for increasing 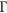 are observed.

Accordingly, the perturbative operator relative to Eq. (16) reads


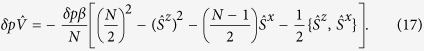


Since 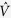 also enjoys complete permutation symmetry, one concludes 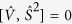, which means 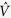 does not produce transitions between different *s*-sector blocks.

Concerning stationary states, the first order correction to eigenvalues 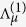 and eigenvectors 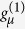, restricted to a single *s*-sector, are obtained using standard perturbation theory^21^,^33^


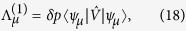



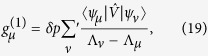


where |*ψ*_*μ*_〉 are the unperturbed eigenvectors of 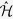. Accordingly, first order correction to the probability to find the system in configuration |*C*_*μ*_〉 is


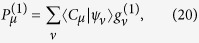


where the unperturbed solutions are evaluated using Eq. (12). Equation (20) emphasizes the role played by the symmetrized operator 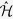 to evaluate the effects caused by topological perturbations: 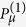 and further perturbative corrections are entirely evaluated using the unperturbed eigenvectors {|*ψ*_*μ*_〉} and eigenvalues 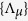, given the perturbation 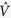. This rationale suits decision making strategies where the concern is the impact of changes to the system topology. In this approach, one avoids computations related to the asymmetric operator 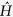, while still benefiting from standard perturbative techniques.

**Figure 9:**
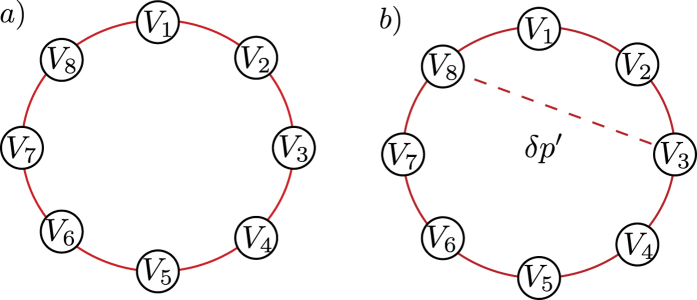
Regular periodic network with *N* = 8 vertices. (**a**) Vertex *V*_*k*_ connects with vertex *V*_*k*+1_ and *V*_*k*−1_ with periodic boundary conditions, *V*_*N*+1_ = *V*_1_ and *V*_0_ = *V*_*N*_. (**b**) Perturbative link addition with probability *δp* = *δp*′/(1 + *δp*′) increases the mean degree *d(k*) by *δp(N* − 2), allowing long range disease transmissions. The graph shows the regular connections for *V*_3_ and an additional connection to *V*_8_.

Complete networks are the simplest instances of a larger set known as regular networks. Another relevant element in the same set is obtained when the connection patterns among vertices are periodic, and are generally employed to describe translation invariant systems. Their eigenvectors are usually categorized in long and short range modes. Since perturbative effects are our main concern here, we now discuss a network with single period, or in the language of condensed-matter, a one-dimensional lattice of size 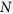 with periodic boundary condition, as Fig. 9(a) illustrates. The adjacency matrix is *A*_*P*_ and the matrix elements are 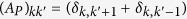, with *V*_0_ = *V*_*N*_ and *V*_*N*+1_ = *V*_1_.

The perturbative scheme to the network topology adds connections with probability 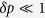 among vertices not previously connected, as shown in Fig. 9(b). The perturbation creates shortcuts throughout the network, favoring rapid disease dispersion, in an attempt to mimic the relevant aspects found in small-world networks^35^. For a single graph realization, translation invariance breaks and the important expression 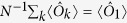 is no longer valid for a general observable 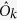. However, for a large ensemble of graphs, the average transition matrix recovers translation invariance. The reasoning behind this claim lies in the fact all vertices would have 2 + *δp(N* − 2) neighbors, on average.

Let *p*_*k,k*′_ = *δp* be the probability to create a single link between *V*_*k*_ and *V*_*k*′_, including nearest-neighbor vertices. The idea is to emphasize the emergence of translation invariance and use the perturbation operator 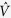 of Eq. (17). Under this assumption, the contributions to the adjacency matrix due to perturbations are *δp*(1 − *δ*_*kk*′_). One must be careful to subtract contributions from links already accounted by *A*_*P*_, resulting in the symmetric time generator 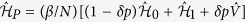. Next, define the effective couplings 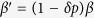 and 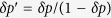, so that


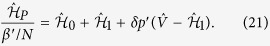


The solution for *δp*′ = 0, the unperturbed system, is obtained using techniques from one-dimensional quantum spinchains, in momentum space^36^. Moreover, total momentum 2*πQ*/*N (Q* = 0, 1, …, *N* − 1) is conserved and serves as a label, breaking 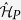 into *N* block-diagonal matrices. For very large *δp*, the topology moves towards the complete network and favors perturbative analysis using Eq. (16) as the unperturbed operator. Therefore, for 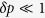 the perturbative regime favors periodic eigenvectors, whereas for 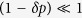, eigenvectors of many-body angular momentum are preferred. The unperturbed eigenvectors and eigenvalues are then used in Eqs (18,19,20) to evaluate the first order perturbative corrections.

## Conclusion

In compartmental approaches to epidemics, the role of fluctuations is underestimated when the population of infected agents is scarce. Disease spreading models using agent-based models are limited to small population sizes due to asymmetric time generators and their large 2^*N*^ dimensions. Our findings show |*P(t*)|^2^ is sufficient to avoid the mathematical hardships that accompany asymmetric operators. The squared norm provides a novel way to obtain stationary states and extremal configurations in general Markov processes, including epidemic models. Once stationary states are secured, the standard Rayleigh-Schrödinger perturbative technique becomes available to epidemics, making use of symmetrized operators and their eigenvalues and eigenvectors. The method paves the way for evaluation of corrections to configuration probabilities caused by perturbations in general networks.

## Additional Information

**How to cite this article**: Nakamura, G. M. *et al*. Efficient method for comprehensive computation of agent-level epidemic dissemination in networks. *Sci. Rep.* **7**, 40885; doi: 10.1038/srep40885 (2017).

**Publisher's note:** Springer Nature remains neutral with regard to jurisdictional claims in published maps and institutional affiliations.

## Supplementary Material

Supplementary Information

## Figures and Tables

**Table 1:** Stationary state.

*μ*	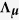	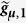
3	0.157199(3)	0.397770(3)
6	0.351413(7)	−0.380366(0)
7	−0.108613(0)	0.834925(5)

Non-vanishing coefficients 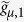 in the SI model with *β*/*N* = 0.1 and *N* = 3. The set 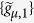 is obtained solving Eq. (12). The coefficients are real and 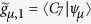, hence, 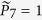.
