# Supplementary material for: Efficient method for comprehensive computation of agent-level epidemic dissemination in networks
Source: Sci Rep. 2017 Jan 20;7:40885. doi: 10.1038/srep40885 (PMC5247741; doi:10.1038/srep40885)
Supplement: Supplementary Information [file srep40885-s1.pdf]

Supplementary Information

Efficient method for comprehensive computation of agent-level epidemic dissemination in networks

Gilberto M. Nakamura,[1, a)] Ana Carolina P. Monteiro,[1] George C. Cardoso,[1] and Alexandre S. Martinez[1]

Universidade de São Paulo, Ribeirão Preto 14040-901, Brazil

[a)] Electronic mail: gmnakamura@usp.br

Appendix A: Example of transition matrix in the SIS model

Let us consider a concrete example of the transition matrix in the SIS model. This laborious work is greatly simplified if one organizes the configuration vectors according to their total number of infected agents. As a result, the example serves as an indicative that other symmetries may also be available. Symmetries are important because they break down $\hat{T}$ into disjoint diagonal blocks, exactly as occurs in Quantum Mechanics, where each block corresponds to an unique set of quantum numbers.

In this example, let $N = 4$ with constant transmission probability β/N, recovery probability γ and the following adjacency matrix:

$$A = \begin{pmatrix} 0 & 1 & 0 & 1 \\ 1 & 0 & 1 & 1 \\ 0 & 1 & 0 & 0 \\ 1 & 1 & 0 & 0 \end{pmatrix}. \tag{A1}$$

The adjacency matrix A is symmetric and represents the simple graph depicted in Fig. 1.

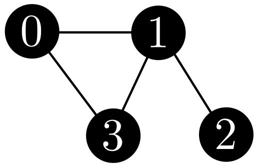

Figure 1. Graph with $N = 4$. Each vertex holds a single agent whose may be either infected or susceptible.

The evaluation of the matrix elements $\langle C_\mu|\hat{T}|C_\nu\rangle$ is straightforward. For instance, consider the application of $\hat{T}$ over $|C_5\rangle$. The off-diagonal recovery operator reads

$$\sum_k \hat{\sigma}_k^- |C_5\rangle = |C_1\rangle + |C_4\rangle, \tag{A2}$$

which in spin notation is

$$\gamma \sum_k \hat{\sigma}_k^- |\uparrow\downarrow\uparrow\downarrow\rangle = \gamma|\uparrow\downarrow\downarrow\downarrow\rangle + \gamma|\downarrow\downarrow\uparrow\downarrow\rangle. \tag{A3}$$

The action of the off-diagonal transmission operator uses the adjacency matrix in Eq. A1,

$$\sum_{k,j} A_{jk}\hat{\sigma}_j^+ \hat{n}_k |C_5\rangle = 2|C_7\rangle + |C_{13}\rangle. \tag{A4}$$

Again, in spin notation the same operation reads

$$\sum_{k,j} A_{jk}\hat{\sigma}_j^{+}\hat{n}_k|\uparrow\downarrow\uparrow\downarrow\rangle = |\uparrow\uparrow\uparrow\downarrow\rangle + |\uparrow\downarrow\uparrow\uparrow\rangle + |\uparrow\uparrow\uparrow\downarrow\rangle. \tag{A5}$$

Therefore,

$$\hat{T}|C_5\rangle = \left(1 - 2\gamma - 3\frac{\beta}{N}\right)|C_5\rangle + \frac{\beta}{N}\left[2|C_7\rangle + |C_{13}\rangle\right] + \gamma\left[|C_1\rangle + |C_4\rangle\right]. \tag{A6}$$

To write down the matrix representation of $\hat{T}$, a convenient order for configuration vectors $|C_\mu\rangle$ is as follows: $[C_0, C_1, C_2, C_4, C_8, C_3, C_6, C_9, C_{12}, C_5, C_{10}, C_7, C_{11}, C_{13}, C_{14}, C_{15}]$. This list orders configurations by the total number of infected agents (ascending) and facilitates the observation that the configurations $[C_1, C_2, C_4, C_8]$ are related by cyclic permutations. The same goes for $[C_3, C_6, C_9, C_{12}]$, $[C_5, C_{10}]$, $[C_7, C_{11}, C_{13}, C_{14}]$. Cyclic permutations are a subset of transformations belonging to the Symmetric Group, which is usually associated with discrete geometric rotations and translations. In the current example, the adjacency matrix is not invariant under cyclic permutations. Using the order proposed in the previous discussion, the matrix representation of $\hat{T}$ is

$$(\hat{T}) = \begin{pmatrix}
1 & \gamma & \gamma & \gamma & \gamma & & & & & & & & & & & \\
 & d_1 & & & & \gamma & & \gamma & & \gamma & & & & & & \\
 & & d_2 & & & \gamma & \gamma & & & & \gamma & & & & & \\
 & & & d_4 & & & \gamma & & \gamma & \gamma & & & & & & \\
 & & & & d_8 & & & \gamma & \gamma & & \gamma & & & & & \\
 & \frac{\beta}{N} & \frac{\beta}{N} & & & d_3 & & & & & & \gamma & \gamma & & & \\
 & & \frac{\beta}{N} & \frac{\beta}{N} & & & d_6 & & & & & \gamma & & & \gamma & \\
 & \frac{\beta}{N} & & & \frac{\beta}{N} & & & d_9 & & & & & \gamma & \gamma & & \\
 & & & & & & & & d_{12} & & & & & \gamma & \gamma & \\
 & & & & & & & & & d_5 & & \gamma & & \gamma & & \\
 & & \frac{\beta}{N} & & \frac{\beta}{N} & & & & & & d_{10} & & \gamma & & \gamma & \\
 & & & & & \frac{\beta}{N} & \frac{\beta}{N} & & & \frac{2\beta}{N} & & d_7 & & & & \gamma \\
 & & & & & \frac{2\beta}{N} & & \frac{2\beta}{N} & & & \frac{2\beta}{N} & & d_{11} & & & \gamma \\
 & & & & & & & & \frac{\beta}{N} & \frac{\beta}{N} & & & & d_{13} & & \gamma \\
 & & & & & & \frac{\beta}{N} & & \frac{2\beta}{N} & & \frac{\beta}{N} & & & & d_{14} & \gamma \\
 & & & & & & & & & & & \frac{2\beta}{N} & \frac{\beta}{N} & \frac{3\beta}{N} & \frac{2\beta}{N} & d_{15}
\end{pmatrix}, \tag{A7}$$

where

$$
\begin{aligned}
d_0 &= 1, & |C_0\rangle &= |\downarrow\downarrow\downarrow\downarrow\rangle, \\
d_1 &= 1 - 2\frac{\beta}{N} - \gamma, & |C_1\rangle &= |\uparrow\downarrow\downarrow\downarrow\rangle, \\
d_2 &= 1 - 3\frac{\beta}{N} - \gamma, & |C_2\rangle &= |\downarrow\uparrow\downarrow\downarrow\rangle, \\
d_4 &= 1 - \frac{\beta}{N} - \gamma, & |C_4\rangle &= |\downarrow\downarrow\uparrow\downarrow\rangle, \\
d_8 &= 1 - 2\frac{\beta}{N} - \gamma, & |C_8\rangle &= |\downarrow\downarrow\downarrow\uparrow\rangle, \\
d_3 &= 1 - 3\frac{\beta}{N} - 2\gamma, & |C_3\rangle &= |\uparrow\uparrow\downarrow\downarrow\rangle, \\
d_6 &= 1 - 2\frac{\beta}{N} - 2\gamma, & |C_6\rangle &= |\downarrow\uparrow\uparrow\downarrow\rangle, \\
d_9 &= 1 - 2\frac{\beta}{N} - 2\gamma, & |C_9\rangle &= |\uparrow\downarrow\downarrow\uparrow\rangle, \\
d_{12} &= 1 - 3\frac{\beta}{N} - 2\gamma, & |C_{12}\rangle &= |\downarrow\downarrow\uparrow\uparrow\rangle, \\
d_5 &= 1 - 3\frac{\beta}{N} - 2\gamma, & |C_5\rangle &= |\uparrow\downarrow\uparrow\downarrow\rangle, \\
d_{10} &= 1 - 3\frac{\beta}{N} - 2\gamma, & |C_{10}\rangle &= |\downarrow\uparrow\downarrow\uparrow\rangle, \\
d_7 &= 1 - 2\frac{\beta}{N} - 3\gamma, & |C_7\rangle &= |\uparrow\uparrow\uparrow\downarrow\rangle, \\
d_{11} &= 1 - \frac{\beta}{N} - 3\gamma, & |C_{11}\rangle &= |\uparrow\uparrow\downarrow\uparrow\rangle, \\
d_{13} &= 1 - 3\frac{\beta}{N} - 3\gamma, & |C_{13}\rangle &= |\uparrow\downarrow\uparrow\uparrow\rangle, \\
d_{14} &= 1 - 2\frac{\beta}{N} - 3\gamma, & |C_{14}\rangle &= |\downarrow\uparrow\uparrow\uparrow\rangle, \\
d_{15} &= 1 - 4\gamma, & |C_{15}\rangle &= |\uparrow\uparrow\uparrow\uparrow\rangle.
\end{aligned}
$$

Appendix B: Generalization for complex epidemic models

The construction of the transition matrix of epidemic models involving $q > 2$ health state per agent follows the same rationale applied to the SIS case. The first step is to assemble the corresponding Hilbert space. After that, local off-diagonal transition operators and local diagonal operators are defined. From these, one assembles the transition matrix. In what follows, we detail each step.

Hilbert space. Let q be the number of distinct health states available for each agent.

Next, let $c_k = 0, 1, \ldots, q-1$ be the variable that represent the health state of the k-th agent, with $k = 0, 1, \ldots, N-1$. For instance, $q = 4$ recovers the health states present in the SEIRS model, with $c_k = 0$ corresponding to susceptible (S); $c_k = 1$ corresponds to the recovered (R); $c_k = 2$ corresponds to the exposed (E); and $c_k = 3$ corresponds to infected (I). Accordingly, the configuration vector for N agents is

$$|C_\mu\rangle = |c_0 c_1 \cdots c_{N-1}\rangle, \tag{B1}$$

$$\mu = c_0 q^0 + c_1 q^1 + \cdots + c_{N-1} q^{N-1}. \tag{B2}$$

The index $\mu = 0, 1, \ldots, q^N - 1$ enumerates all possible configurations of N agents. These definitions are slightly different from the ones introduced for the SIS model, where we reinforced the analogy with 1/2-spin states and 1/2-spin operators. Nevertheless, both formulations are equivalent if we change $\sigma_k =\uparrow$ to $c_k = 1$ and $\sigma_k =\downarrow$ to $c_k = 0$.

Local operators. For $q = 2$ health states per agent, local raising and lowering operators ($\hat{S}_k^{+}$) are intuitive choices to produce off-diagonal transitions. However, the same choice for $q > 2$ implies that the action of angular operators also multiplies the resulting vector by scalar numbers $\sqrt{l(l+1) - m(m \pm 1)}$, with $l = q/2, q/2 - 1, \ldots,$ $q \geq 0$, and $m = -l, -l + 1, \ldots, l$. Therefore any transition matrix employing $\hat{S}_k^{\pm}$ would have to taken into account the conserved quantum number l, which implies the introduction of effective transition probabilities for each l-sector. This is clearly problematic as one would have to give up the configuration vectors $|C_\mu\rangle$.

A much more elegant solution to address local operators makes use of the local $q \times q$ Weyl matrices, $\hat{E}_k^{x_2 x_1}$, with $k = 0, 1, \ldots, N-1$ and $x_{1,2} = 0, 1, \ldots, q-1$. The matrix elements of $\hat{E}_k^{x_2 x_1}$ are essentially null except for a single entry, $(\hat{E}_k^{x_2 x_1})_{ij} = \delta_{i,x_2}\delta_{j,x_1}$. This property of the Weyl matrices allows for a simple interpretation: $\hat{E}_k^{x_2 x_1}$ changes c_k from $c_k = x_1$ to $c_k = x_2$. For instance, the transition from susceptible to exposed ($S \to E$) in the SEIRS model occurs due to $\hat{E}_k^{20}$, *i.e.*,

$$\hat{E}_k^{20} |c_0 c_1 \cdots 0_k \cdots c_{N-1}\rangle = |c_0 c_1 \cdots 2_k \cdots c_{N-1}\rangle. \tag{B3}$$

The diagonal operators are also obtained after the Weyl matrices $\hat{E}_k^{yx}$ when both the incoming index x equals the outgoing index y. According to this definition,

$$\hat{E}_k^{xx} |C_\mu\rangle = \delta_{x,c_k} |C_\mu\rangle, \tag{B4}$$

so that $\hat{E}_k^{xx}$ only measures whether the k-th agent is in the state x or not.

In summary, $\hat{E}_k^{x_2 x_1}$ produce operators that modify configuration vectors $|C_\mu\rangle$ but do not multiply them by scalars that depends on the quantum number l. This property makes them suitable candidates to build the transition matrix of general epidemic models.

Transition matrix. The off-diagonal components of the transition matrix contemplates operators involving either only one agent or two distinct agents. The transition $I \to R$ is an example of single agent transition, as it only modifies the health state of a single agent. These transitions are trivially expressed by single Weyl matrices $\hat{E}_k^{yx}$, each with corresponding probabilities D^{yx}. Now, in two-agent transitions the k-th agent provokes a state change in the j-th agent. This kind of transition requires explicitly the adjacency matrix A_{jk} to check whether they are connected. Therefore, two-agent off-diagonal transitions occurs due to $A_{jk}\hat{E}_j^{y_2 y_1}\hat{E}_k^{x_2 x_1}$, with corresponding probabilities $\Gamma_{x_2 x_1}^{y_2 y_1}$. In most epidemic models, it is assumed the transmission of a pathogen does not changes the state of the infectious agent. Therefore, it is safe to assume that $x_2 = x_1$, *i.e.*, an infected agent remains infected after transmitting the pathogen one susceptible agent.

The difficult part is to evaluate the diagonal components: the probability that a given configuration $|C_\mu\rangle$ remains unchanged after the application of $\hat{T}$. However, this is achieved by simply considering the probabilities that no transitions occur. For example, if we consider *only* single agent transitions, then the operator that tell us the chance to remain unchanged is

$$\mathbb{1} - \sum_k \sum_{x,y=0}^{q-1} D^{yx}\hat{E}_k^{xx} = \mathbb{1} - \sum_k \sum_x \hat{E}_k^{xx}(\sum_y D^{yx}). \tag{B5}$$

Inclusion of two-agents transitions is straightforward and shown below.

Therefore, the complete transition matrix is obtained by summing both off-diagonal and diagonal contributions:

$$\hat{T} = \mathbb{1} - \sum_{k,j=0}^{N-1} \sum_{a,b,c=0}^{q-1} \Gamma_{cc}^{ba} A_{jk} \left(\hat{E}_j^{bb} - \hat{E}_j^{ba}\right)\hat{E}_k^{cc} - \sum_{k=0}^{N-1} \sum_{a,b=0}^{q-1} D^{ba}\left(\hat{E}_k^{aa} - \hat{E}_k^{ba}\right). \tag{B6}$$

In this formulation, it is easy to verify the summation of both diagonal and off-diagonal transition for a single configuration vector $|C_\nu\rangle$ conserves unitary probability, $\sum_\mu (\hat{T})_{\mu\nu} = 1$. Again, we use the SEIRS model as working example by setting $q = 4$. The only two-agent transition available describes an infected agent transmits the pathogen to a susceptible one ($SI \to EI$), with probability Γ_{33}^{20}. The non-vanishing single agent transitions occur with probabilities D^{32} and corresponds to $E \to I$, D^{13} to $I \to R$, and D^{01} to $R \to I$.

Appendix C: Observables

The equation

$$-\frac{d}{dt}|P(t)\rangle = \hat{H}|P(t)\rangle \tag{C1}$$

provides also the necessary information to evaluate the dynamical equations for statistics, including the average number of infected agents $\langle n(t)\rangle = \sum_\mu \langle C_\mu| \sum_k \hat{n}_k |P(t)\rangle$. For the SIS model, the application of the operator $\hat{n} = \sum_k \hat{n}_k$ on Eq. (C1), followed by summation over index μ, results in

$$\begin{aligned} -\frac{d}{dt}\langle n\rangle = &+ \frac{\beta}{N}\sum_{\mu\nu}\sum_{ijk} A_{ij} P_\nu \langle C_\mu|\hat{n}_k(1-\hat{n}_i)\hat{n}_j - \hat{n}_k\hat{\sigma}_i^+\hat{n}_j|C_\nu\rangle + \\ &+ \gamma \sum_{\mu\nu}\sum_{ik} P_\nu \langle C_\mu|\hat{n}_k(\hat{n}_i - \hat{\sigma}_i^-)|C_\nu\rangle. \end{aligned} \tag{C2}$$

For the complete graph, $A_{ij} = 1 - \delta_{ij}$, the non-vanishing matrix elements are

$$\langle C_\mu| \sum_{k=1}^{N} \hat{\sigma}_k^+ |C_\nu\rangle = \delta_{n_\mu, n_\nu+1}(N - n_\nu), \tag{C3a}$$

$$\langle C_\mu| \sum_{k=1}^{N} \hat{\sigma}_k^- |C_\nu\rangle = \delta_{n_\mu, n_\nu-1} n_\nu, \tag{C3b}$$

where $n_\mu = \langle C_\mu| \sum_k \hat{n} |C_\mu\rangle$ stands for the number of infected agents in the configuration C_μ. Substituting Eqs. (C3a) and (C3b) in Eq. (C2), one obtains

$$\begin{aligned} -\frac{d}{dt}\langle n\rangle = &+ \frac{\beta}{N}\sum_\nu P_\nu n_\nu \left[n_\nu(N-n_\nu) - (n_\nu+1)(N-n_\nu)\right] + \\ &+ \gamma \sum_\nu P_\nu n_\nu \left[n_\nu - (n_\nu - 1)\right] \\ \frac{d}{dt}\langle n\rangle = &\ \frac{\beta}{N}\sum_\mu P_\mu n_\mu \left(N - n_\mu\right) - \gamma\langle n\rangle. \end{aligned} \tag{C4}$$

The compartmental equation is derived by assuming that there is no dispersion, $\sum_\mu P_\mu n_\mu^2 = \langle n^2\rangle \approx \langle n\rangle^2$:

$$\frac{d\rho}{dt} = \beta\rho\left(1-\rho\right) - \gamma\rho, \tag{C5}$$

with $\rho = \langle n\rangle/N$. A more familiar expression is obtained by multiplying the right-hand side of Eq. (C5) by the ratio $\langle k\rangle/N$ between the average degree $\langle k\rangle$ and N. Since $\langle k\rangle = N-1$ for the complete graph, the ratio converges to unity for large N:

$$\frac{d\rho}{dt} = \beta\left(\frac{\langle k\rangle}{N}\right)\rho\left(1-\rho\right) - \gamma\rho, \tag{C6}$$

A similar compartmental equation is obtained in random networks, with the substitution $\beta \to \beta p$, where p is the uniform probability that a link exists between any two distinct vertices. The grounds for this claim can be tracked down to Eq.(C1). The disease spreading takes place in an ensemble of graphs, each with N vertices, with the same initial conditions. The size of the ensemble is M and each graph is a random graph, in which links are distributed according to the independent and uniform probability p. In this case, the average $\langle A_{ij}\rangle_E = p$, where the subindex E means the average is taken over the ensemble of graphs. Since the initial conditions are the same, the average time evolution within the ensemble is simply

$$-\frac{d}{dt}|P(t)\rangle = \frac{1}{M}\sum_{m=1}^{M}\hat{H}^{(m)}|P(t)\rangle, \tag{C7}$$

where $\hat{H}^{(m)}$ is the time generator corresponding to the m-th random graph. This is equivalent to average the operator $\hat{H}$, *i.e.* $A_{ij} \to p(1-\delta_{ij})$. By following the same steps as before, one arrives at

$$\frac{d\rho}{dt} = (p\beta)\rho(1-\rho) - \gamma\rho. \tag{C8}$$

Equation (C8) assumes a more familiar expression by multiplying the right-hand side by N/N. Since $\langle k\rangle = pN$, Eq. (C8) reads:

$$\frac{d\rho}{dt} = \beta\left(\frac{\langle k\rangle}{N}\right)\rho(1-\rho) - \gamma\rho. \tag{C9}$$

If the epidemic takes places in more complex graphs, the graph structure is taken into account as follows. Let $n_{\mu i} = \langle C_\mu|\hat{n}_i|C_\mu\rangle$. Again, only two matrix elements are non-diagonal in Eq. (C2),

$$\langle C_\mu|\hat{\sigma}_i^+|C_\nu\rangle = \delta_{n_\mu, n_\nu+1}(1-n_{\nu i}), \tag{C10a}$$

$$\langle C_\mu|\hat{\sigma}_i^-|C_\nu\rangle = \delta_{n_\mu, n_\nu-1}n_{\nu i}. \tag{C10b}$$

Note that in the expressions above, the total number of infected elements still appear in the Kronecker delta, which simplifies Eq. (C2):

$$\frac{d}{dt}\langle n\rangle = \frac{\beta}{N}\left[\sum_\nu\sum_{i,j}^{N}P_\nu A_{ij}(1-n_{\nu i})n_{\nu j}\right] - \gamma\langle n\rangle. \tag{C11}$$

Introduced by Van Mieghem[1], the N-intertwined mean-field approximation is obtained by assuming $n_i \equiv \sum_\mu P_\mu n_{\mu i}$ are independent and uncorrelated variables, $\sum_\mu P_\mu n_{\mu i}n_{\mu j} \approx n_i n_j$.

Under this approximation, Eq. (C11) is rewritten as a system of N independent equations,

$$\frac{dn_i}{dt} = \frac{\beta}{N}\left[\sum_j^N A_{ij}(1-n_i)n_j\right] - \gamma n_i, \tag{C12}$$

with $i = 1, \dots, N$.

Appendix D: Continuous spectral equation

In the $N \gg 1$ regime, the eigenspectrum becomes dense and it is convenient to analyse

$$\sum_\mu \left(\frac{1}{2}\frac{d}{dt} + \Lambda_\mu\right)|g_\mu(t)|^2 = 0, \tag{D1}$$

using the continuous variable Λ. Let $\rho(\Lambda)$ be the density of states between Λ and $\Lambda + \delta\Lambda$. In addition, consider the real spectral functions $\eta_1(\Lambda, t)$ and $\eta_2(\Lambda, t)$ so that $g_\mu(t) \to \eta_1(\Lambda, t) + \imath\,\eta_2(\Lambda, t)$, with squared norm $\eta^2(\Lambda, t) \equiv \eta_1^2(\Lambda, t) + \eta_2^2(\Lambda, t)$. Since the time evolution of $|P(t)|^2$ is deterministic, it is convenient to define the functional $S[P]$ over a time interval $t_1 - t_0$,

$$S[P] = \int_{t_0}^{t_1} dt\, |P(t)|^2, \tag{D2}$$

with

$$|P(t)|^2 = \int_{-\infty}^{\infty} d\Lambda \rho(\Lambda)\eta^2(\Lambda, t). \tag{D3}$$

Equation (D2) suggests the interpretation of $S[P]$ as the system action. Let the underlying network link distribution be a continuous function of the real parameter q. This assumption is reasonable for random or regular networks, however, it is not necessarily valid for more complex networks. Next, one considers virtual variations δq to q, which produce the change $\delta\rho(\Lambda)$ in the density of states. According to Eq. (D1), the continuous variables Λ and real functions ρ and η satisfy the following spectral equation:

$$\int_{-\infty}^{\infty} d\Lambda \left(\frac{1}{2}\frac{\partial}{\partial t} + \Lambda\right)\rho(\Lambda)\eta^2(\Lambda, t) = 0. \tag{D4}$$

REFERENCES

[1] P. Van Mieghem, "The n-intertwined sis epidemic network model," Computing **93**, 147–169 (2011).
